# A new cockroach (Blattodea, Corydiidae) with pectinate antennae from mid-Cretaceous Burmese amber

**DOI:** 10.3897/zookeys.1060.67216

**Published:** 2021-09-24

**Authors:** Guanyu Chen, Lifang Xiao, Junhui Liang, Chungkun Shih, Dong Ren

**Affiliations:** 1 College of Life Sciences and Academy for Multidisciplinary Studies, Capital Normal University, 105 Xisanhuanbeilu, Haidian District, Beijing 100048, China Capital Normal University Beijing China; 2 Tianjin Natural History Museum, 31 Youyi Road, Hexi District, Tianjin, 300203, China Tianjin Natural History Museum Tianjin China; 3 Department of Paleobiology, National Museum of Natural History, Smithsonian Institution, Washington, DC, 20013–7012, USA National Museum of Natural History, Smithsonian Institution Washington United States of America

**Keywords:** Convergent evolution, Myanmar, new genus, new species, pectinate antenna, sexual dimorphism, systematic palaeoentomology

## Abstract

A new species of fossil cockroach, *Fragosublattapectinata***gen. et sp. nov.**, is described from mid-Cretaceous Burmese amber. The new species is assigned to the family Corydiidae based on the following combination of characters: pronotum with tubercles, tegmina obovate with smallish anal region and spinules on the antero-ventral margin of the front femur (type C1). The new species is the second reported cockroach with ramified antennae. This finding broadens the diversity of Blattodea in mid-Cretaceous Burmese amber and provides further evidence of convergent evolution for antennal structures among different insect lineages.

## Introduction

Blattodea is an order of insects consisting of cockroaches and termites (Inward et al. 2007; Zhao et al. 2019). Up to date, about 5000 extant cockroach species and 1500 fossil species have been documented (Liang et al. 2019; Li et al. 2020).

Diverse insects have been documented from the mid-Cretaceous Burmese (Myanmar) amber recently (Ross 2020, 2021). An ecosystem with a humid climate in the mid-Cretaceous enriched the diversity of cockroach species (Liang et al. 2019). Up to now, 11 families, 28 genera and 36 species of cockroaches in Burmese amber have been documented as shown in Table 1 (Ross 2021). However, only two extinct species of Corydiidae have been reported in Burmese amber so far. The specimens in Burmese amber give us an opportunity to better understand the morphological characters of ancient insects.

**Table 1.** Records of cockroaches described in Burmese amber.

Family	Species	Reference
Blattulidae	* Huablattula hui *	Qiu et al. 2019a
	* Huablattula jiewenae *	Qiu et al. 2019a
Mesoblattinidae	* Spinaeblattina myanmarensis *	Hinkelman 2019
	* Mesoblatta maxi *	Hinkelman and Vršanská 2020
Raphidiomimidae	* Raphidiomimula burmitica *	Grimaldi and Ross 2004
Liberiblattinidae	* Spongistoma angusta *	Sendi et al. 2020a
	* Stavba babkaeva *	Vršanský et al. 2019
	* Stavba vrsanskyi *	Chen et al. 2020
	* Stavba jarzembowskii *	Li et al. 2020
Olidae	* Ol xiai *	Vršanský and Wang 2017
Alienopteridae	* Vzrkadlenie miso *	Sendi et al. 2020a
	* Formicamendax vršanskýi *	Hinkelman 2020
	* Teyia branislav *	Vršanský et al. 2018a
	* Teyia huangi *	Vršanský et al. 2018a.
	* Meilia jinghanae *	Vršanský et al. 2018a
	* Caputoraptor vidit *	Vršanský et al. 2018a
	* Alienopterix ocularis *	Vršanský et al. 2018a
	* Alienopterix smidovae *	Vršanský et al. 2021
	* Alienopterix mlynskyi *	Vršanský et al. 2021
	* Nadveruzenie postava *	Vršanský et al. 2021
Umenocoleidae	* Jantaropterix ellenbergeri *	Mlynský et al. 2019
	* Cratovitisma bechlyi *	Podstrelená and Sendi 2018
	* Perspicuus pilosus *	Koubová and Mlynský 2020
	* Perspicuus vršanský *	Koubová and Mlynský 2020
	* Antophiloblatta hispida *	Sendi et al. 2020a
Blattidae	* Cretaperiplaneta kaonashi *	Qiu et al. 2020
	* Balatronis cretacea *	Šmídová and Lei 2017
	* Bubosa poinari *	Šmídová. 2020
	* Spinka fussa *	Vršanský et al. 2018b
Corydiidae	* Nodosigalea burmanica *	Li and Huang 2018
	* Magniocula apiculata *	Qiu et al. 2019b
Nocticolidae	* Mulleriblattina bowangi *	Sendi et al. 2020b
	* Crenocticola svadba *	Sendi et al. 2020b
	* Crenocticola burmanica *	Li and Huang 2019
Manipulatoridae	* Manipulator modificaputis *	Vršanský and Bechly 2015
Incertae sedis	* Cercoula brachyptera *	Li and Huang 2021

Antennae of insects harbor the functions of smell, taste and other senses (Schneider 1964). Some insects have evolved ramified antennae, ranging from forms that are pectinate or bipectinate to plumose (Gao et al. 2016). As documented in the fossil record, 26 insect species in six orders, mostly males, have preserved ramified antennae, e.g., *Atefiarasnitsyni* (Hymenoptera), *Palaeopsilotretaburmanica* (Trichoptera), *Vitimopsychepectinella* (Mecoptera), *Olxiai* (Blattodea), *Oligopsychopsispenniformis* (Neuroptera), *Cerophytumalbertalleni* (Coleoptera), as summarized in Table 2. Nevertheless, cockroaches with ramified antennae are very rare, with only one reported species (*Olxiai*, male) in Olidae having bipectinate antennae (Vršanský and Wang 2017).

**Table 2.** Ramified antennal types of different insect orders in the Cretaceous.

Order	Antennal type	Family	Species	Locality	Reference
Mecoptera	pectinate	Mesopsychidae	* Vitimopsyche pectinella *	China	Gao et al. 2016
	pectinate	Mesopsychidae	* Vitimopsyche kozlovi *	China	Ren et al. 2009
Trichoptera	bipectinate	Calamoceratidae	* Bipectinata orientalis *	Myanmar	Wichard et al. 2020
	bipectinate	Odontoceridae	* Palaeopsilotreta cretacea *	Myanmar	Wichard et al. 2020
	bipectinate	Odontoceridae	* Palaeopsilotreta burmanica *	Myanmar	Wichard et al. 2020
	bipectinate	Odontoceridae	* Palaeopsilotreta xiai *	Myanmar	Wichard et al. 2020
	bipectinate	Incertae sedis	* Cathayamodus fournieri *	China	Gao et al. 2016
Hymenoptera	pectinate	Megalodontesidae	* Jibaissodes peichenae *	China	Wang et al. 2019
	plumose	Megalodontesidae	* Jibaissodes bellus *	China	Gao et al. 2016
	flabellate	Incertae sedis	* Atefia rasnitsyni *	Brazil	Krogmann et al. 2013
Coleoptera	pectinate	Cerophytidae	* Cerophytum albertalleni *	Myanmar	Yu et al. 2019
	pectinate	Brachypsectridae	* Vetubrachypsectra burmitica *	Myanmar	Qu et al. 2019
	pectinate	Lycidae	* Prototrichalus sepronai *	Myanmar	Molino-Olmedo et al. 2020
	pectinate	Cantharidae	* Burmomiles willerslevorum *	Myanmar	Fanti et al. 2018
	pectinate	Cantharidae	* Sanaungulus curtipennis *	Myanmar	Fanti et al. 2018
	pectinate	Cantharidae	* Sanaungulus ghitaenoerbyae *	Myanmar	Fanti et al. 2018
Neuroptera	bipectinate	Incertae sedis	* Oligopsychopsis penniformis *	Myanmar	Chang et al. 2017
	bipectinate	Kalligrammatidae	* Burmogramma liui *	Myanmar	Liu et al. 2018
	bipectinate	Kalligrammatidae	* Burmopsychops labandeirai *	Myanmar	Liu et al. 2018
	bipectinate	Kalligrammatidae	* Cretogramma engeli *	Myanmar	Liu et al. 2018
	bipectinate	Kalligrammatidae	* Oligopsychopsis grandis *	Myanmar	Liu et al. 2018
	pectinate	Dilaridae	* Cretanallachius magnificus *	Myanmar	Huang et al. 2015
	pectinate	Dilaridae	* Cretadilar olei *	Myanmar	Makarkin 2016
	pectinate	Dilaridae	* Burmopsychops groehni *	Myanmar	Makarkin 2016
	pectinate	Dilaridae	* Cretodilar burmanus *	Myanmar	Liu et al. 2016
Blattodea	bipectinate	Olidae	* Ol xiai *	Myanmar	Vršanský and Wang 2017

Herein, we describe a new genus and species, *Fragosublattapectinata* gen. et sp. nov., assigned to Corydiidae. This new finding broadens the diversity of Blattodea in mid-Cretaceous Burmese amber, clarifies the varieties of their antennal morphology, and suggests a potential sexual dimorphism for these cockroaches.

## Material and methods

The type specimen was collected from deposits in the Hukawng Valley of Kachin in northern Myanmar, approximately 100 km southwest of the village of Tanai. The age of Myanmar amber is documented as 98.79±0.62 Mya, in the mid-Cretaceous (Grimaldi and Ross 2017). Myanmar amber pieces have preserved abundant specimens of plants, insects and other invertebrates. The latest comprehensive list of insect taxa from Myanmar amber comprises 28 orders, 421 families, 975 genera and 1383 species (Ross 2020, 2021). The type specimen is housed in the Key Laboratory of Insect Evolution and Environmental Changes, College of Life Sciences and Academy for Multidisciplinary Studies, Capital Normal University, Beijing, China (CNUB; Dong Ren, Curator).

The new specimen was examined and photographed using a Leica M205C dissecting microscope with a Leica DFC450 digital camera system. The detailed and enlarged photos were taken by using a Nikon SMZ 25 microscope with a Nikon DS-Ri 2 digital camera system. Cool white transmitted light from microscope’s LED illuminators passed through the specimen from the top, and cool white light, emitted from double optical fibers, irradiated the specimen from two sides simultaneously. Line drawings were prepared by using Adobe Illustrator CC and Adobe Photoshop CS5 graphics software.

Morphological terminology largely follows Roth (2003); venational terms follow Snodgrass (1935), with further interpretations by Smart (1951) and Li and Wang (2017) as a frame of reference.

## Systematic palaeoentomology

**Order Blattodea Brunner von Wattenwyl, 1882**

### Family Corydiidae Saussure & Zehntner, 1893

#### Genus Fragosublatta

Taxon classificationAnimaliaBlattodeaCorydiidae

Chen, Shih & Ren gen. nov.

012A751F-4CA3-5EBA-8E5D-9E9B86B95B95

http://zoobank.org/97CB1AFA-A97C-4A12-AA36-CD3070D3F840

##### Diagnosis.

(male only). Sc field narrow (about a third of the width of the R region) with Sc short and branched. CuA almost straight with comb-like branches. CuP sharply curved. The first and the second hind tarsomeres with no plantulae but with spines. Cercus monoliform.

##### Etymology.

*Fragosublatta* is a combination of *fragosus* (Latin for fractured), referring to the fractured pronotum, and the generic name of *Blatta*. Gender is feminine.

##### Remarks.

The new species is assigned to the family Corydiidae based on these characters: pronotum with tubercles, tegmina obovate with smallish anal region and spinules on the antero-ventral margin of the front femur (type C1). The new genus is differentiated from other extinct genera mainly by the forewing and legs: CuA with comb-like branches and the first and the second hind tarsomeres apparently lacking plantulae but with spines. Besides, the subgenital plate of the new species is almost symmetrical, which is similar to *Nodosigaleaburmanica* (Li & Huang, 2018), but the new species has comb-like CuA branches to justify the erection of a new genus.

#### Fragosublatta pectinata

Taxon classificationAnimaliaBlattodeaCorydiidae

Chen, Shih & Ren sp. nov.

31097BAA-FCE4-54B2-A26F-484DCFDE8911

http://zoobank.org/0576681A-20FA-46D6-8ED9-6003EA0F69DB

Figs 1 , 2 , 3 , 4

##### Type material.

***Holotype***: CNU-BLA-MA2015001, a male specimen. The specimen was preserved in amber at an angle. Most of the insect body parts are preserved, but major parts of the head and all left tibiae and tarsi are missing. The pronotum and the left forewing are fractured.

##### Locality and horizon.

Hukawng Valley, Kachin State, northern Myanmar; lowermost Cenomanian, mid-Cretaceous.

##### Diagnosis.

As for the genus due to monotype.

##### Description.

**Figure 1.**
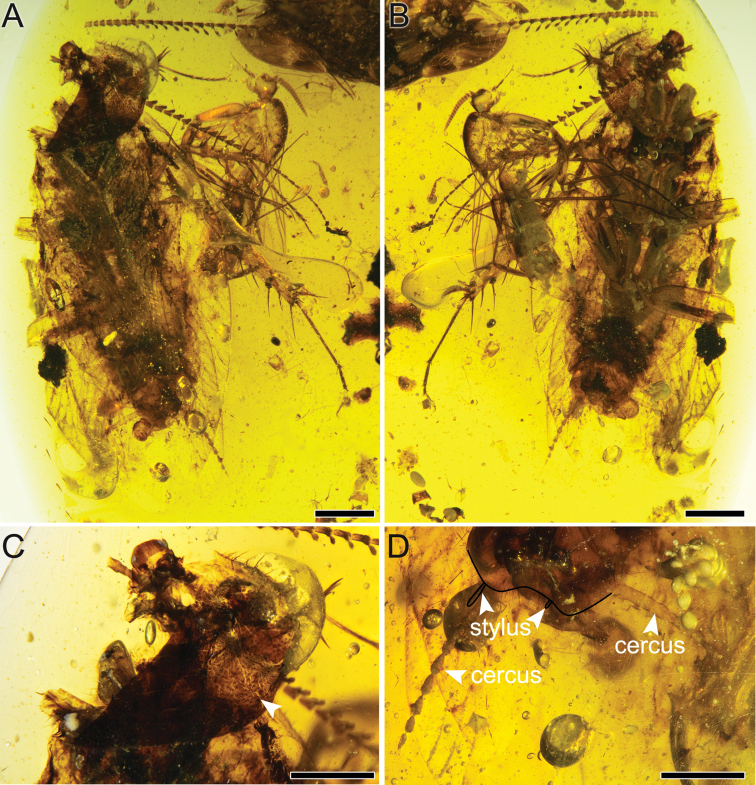
Holotype of *Fragosublattapectinata* gen. et sp. nov. CNU-BLA-MA2015001 **A** photograph of habitus in dorsal view **B** photograph of habitus in ventral view **C** photograph of the pronotum, with arrowhead indicating the tubercles **D** photograph of the moniliform cercus and asymmetrical stylus. Scale bars: 1.0 mm (**A, B**), 0.2 mm (**C, D**).

**Figure 2.**
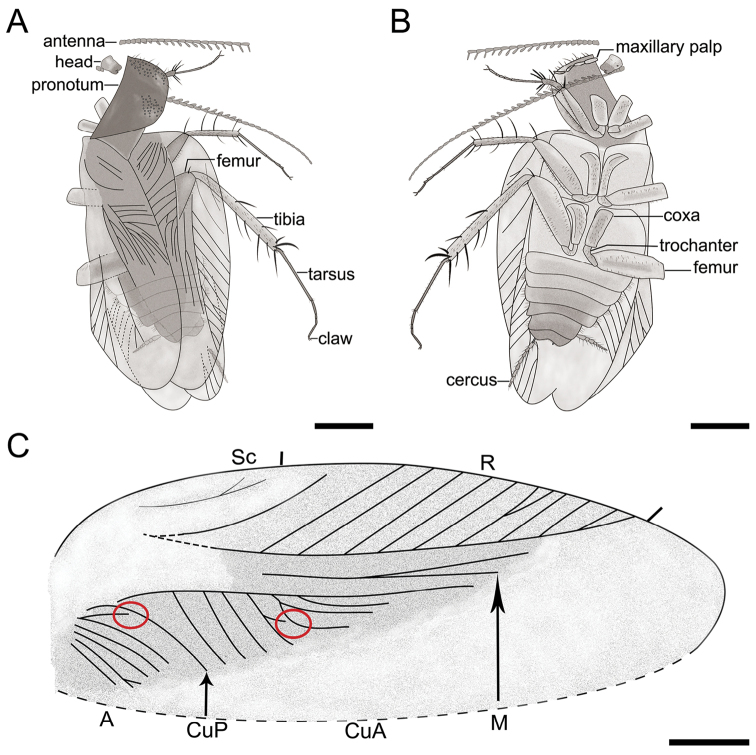
Holotype of *Fragosublattapectinata* gen. et sp. nov. CNU-BLA-MA2015001 **A** line drawing in dorsal view **B** line drawing in ventral view **C** line drawing of the right forewing, with circles indicating the incomplete veins. Scale bars: 1.0 mm (**A, B**), 0.5 mm, (**C**).

**Figure 3.**
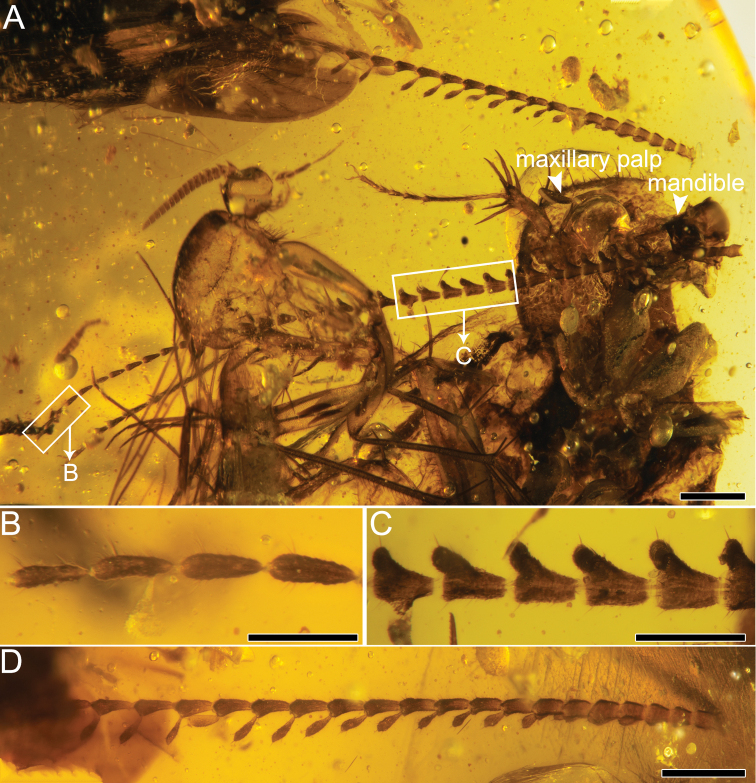
Holotype of *Fragosublattapectinata* gen. et sp. nov. CNU-BLA-MA2015001 **A** photograph of the two antennae, with arrowheads indicating the maxillary palp and the mandible **B** the apical section of the longer antenna **C** the medial section of the longer antenna **D** photograph of the shorter antenna. Scale bars: 0.5 mm (**A**), 0.1 mm (**B, C**), 0.25 mm (**D**).

Medium-sized brown cockroach, body narrow and flattened, overall body length 8.21 mm/width 2.97 mm (Fig. 1A, B). Major parts of head not preserved. Eyes and labial palps invisible. Mandibles with two sharp teeth preserved (Fig. 3A). Only four maxillary palps preserved (total length 1.02 mm), with terminal palpomere oval in shape. Sensilla on palps dense and small, < 0.01 mm wide. Both antennae detached from head and missing some antennomeres (Fig. 2A, B); antennae with 19 and 40 antennomeres respectively; length of antennae slightly shorter than forewing length; both antennae with comb-like extensions at end of each flagellomere. Basal flagellomeres simple, thick and short, medial 20 successive flagellomeres pectinate and apical 13 flagellomeres simple (Fig. 3). Longest comb-like extension of pectinate flagellomeres 0.19 mm. Antennomeres roundish to cylindrical with widest base of 0.13 mm. Pronotum (length 2.15 mm/width 1.84 mm, as preserved) with dense tubercles, nearly vaulted (Fig. 1C), partly sclerotized and melanized, anterior margin covered with obvious hairs. Scutellum distinct, long and wide (ca 0.75/ca1.18 mm).

Forewing obovate, overlapping each other and completely covering abdomen. Left forewing overlapping right forewing. Right forewing 7.7 mm long, anterior margin arched, apex rounded (Fig. 2C). Right forewing costa 2.13 mm long. Sc field narrow, slightly curved, dichotomized with two veins not meeting margin, occupying about one third of forewing length. R regularly branched. M with only two branches. CuA almost straight, posterior-most veins comb-like, up to nine veins preserved. CuP sharply curved. Most of clavus area sclerotized, anal area obviously smallish, with seven veins. Left forewing 7.37 mm long, damaged basally. R with six visible branches. M with only two branches preserved. CuA richly branched with distinct intercalary veins. CuP simple, probably with only two and relatively straight A veins. Hind wing membranous, transparent. R branched, with 6–7 visible veins, reaching wing margin.

**Figure 4.**
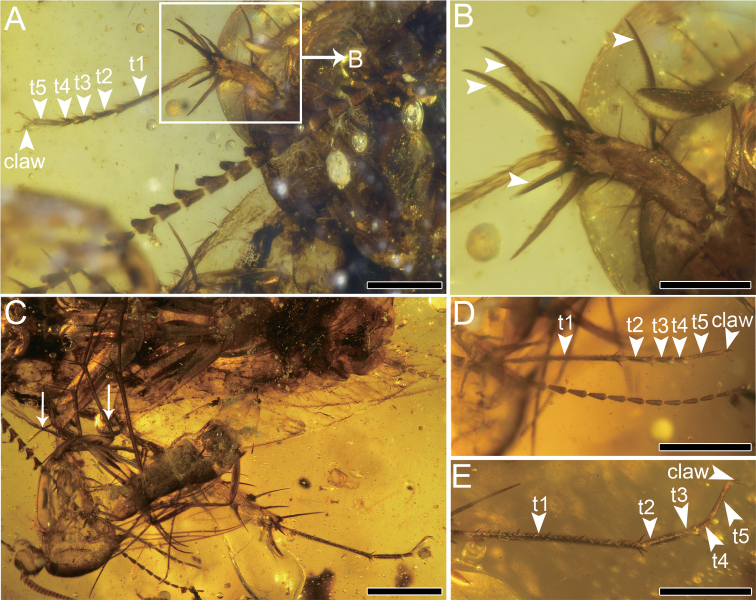
Holotype of *Fragosublattapectinata* gen. et sp. nov. CNU-BLA-MA2015001 **A** photograph of the foreleg **B** details of the foretibia spurs, with arrowheads indicating the serration **C** photograph of the midleg and hind leg, with arrowheads indicating the terminal spines **D** photograph of the midtarsus **E** photograph of the hind tarsus. Scale bars: 0.5 mm (**A, C**), 0.25 mm (**B, D, E**).

From fore legs to hind legs gradually stronger. Fore coxa short and wide (length 0.76 mm/width 0.37 mm). Femur with carination, 1.15 mm long and 0.28 mm wide, antero-ventral margin of fore femur with even spinules (type C1 according to Roth 2003), terminal spine 0.36 mm long, slightly curved (Fig. 4A). Tibia (length 0.73 mm/width 0.17 mm) typical in Corydiidae, with long spines, most of spines with serrations (Fig. 4B). Tarsi five-segmented (length 0.76/0.18/0.14/0.13/0.23 mm), with a total of 1.44 mm long and 0.04 mm wide. Claw symmetrical (Fig. 4A), strong, 0.18 mm long, arolium absent. Mid coxa with carination, 1.04 mm long and 0.2 mm wide. Trochanter comparatively longer (length 0.39 mm). Femur 1.87 mm long and 0.44 mm wide with two rows of spinules. Terminal spine not curved distinctly, 0.48 mm long (Fig. 4C). Tibia approximately as long as femur, 1.51 mm long and 0.17 mm wide, with seven spines. Tarsi 2.03 mm long and 0.05 mm wide, first tarsomere longest (length 0.68 mm), terminal tarsomere with symmetrical claws (length 0.13 mm). Hind coxa 1.2 mm long with obvious carination, narrowing from top to bottom. Hind trochanter 0.4 mm long and 0.6 mm wide. Femur strong (length 2.03 mm/width 0.60 mm) with terminal spine 0.29 mm long (Fig. 4C). Tibia longer (length 3.08 mm/width 0.28 mm) with at least 10 spurs. Tarsi five-segmented (tarsomeres 1–5 lengths 0.82–0.39–0.37–0.36–0.41 mm) but narrow (width 0.07 mm). Plantulae present at four proximal tarsomeres in fore and mid tarsi, which also exist in third and fourth tarsomeres of hind leg. First and second hind tarsomeres apparently have spines, but lack plantulae (Fig. 4A, D, E). Six sternites visible on abdomen, with sparse chaetae. Cercus moniliform, completely preserved with up to 0.23 mm long sensilla chaetica, divided into eight cercomeres on left (ca 1.51 mm) and nine on right (ca 1.73 mm), basally thicker and apically narrower (Fig. 1D). Hind margin of subgenital plate convex, setose, with a wide concave incision medially. Styli asymmetrical, left stylus longer (length 0.35 mm) than right stylus (0.16 mm long). Both styli unsegmented.

##### Etymology.

The name *pectinata* is derived from the Latin word of *pectinatus* referring to the pectinate antennae.

##### Remarks.

The antennae are detached from the head of *Fragosublattapectinata* gen. et sp. nov., but the basal antennomeres of both antennae are close to the head (Fig. 3A). As shown in Figs 1B and 2B, the length of the left antennae, as preserved, is slightly shorter than the forewing length, which is consistent with the length ratios of the antennae/forewing for many documented fossil cockroaches (Liang et al. 2019). Therefore, we have high confidence that these two antennae belong to *Fragosublattapectinata* gen. et sp. nov. based on these observations. Besides, there are two syninclusions in this amber piece, including a MycetophiloideaDiptera and a Hemiptera ‘Homoptera’ (suspected) close to the hind legs of the new species. Due to poor preservation, we cannot identify the detailed taxonomic classification for these two syninclusions.

## Discussion

**Figure 5.**
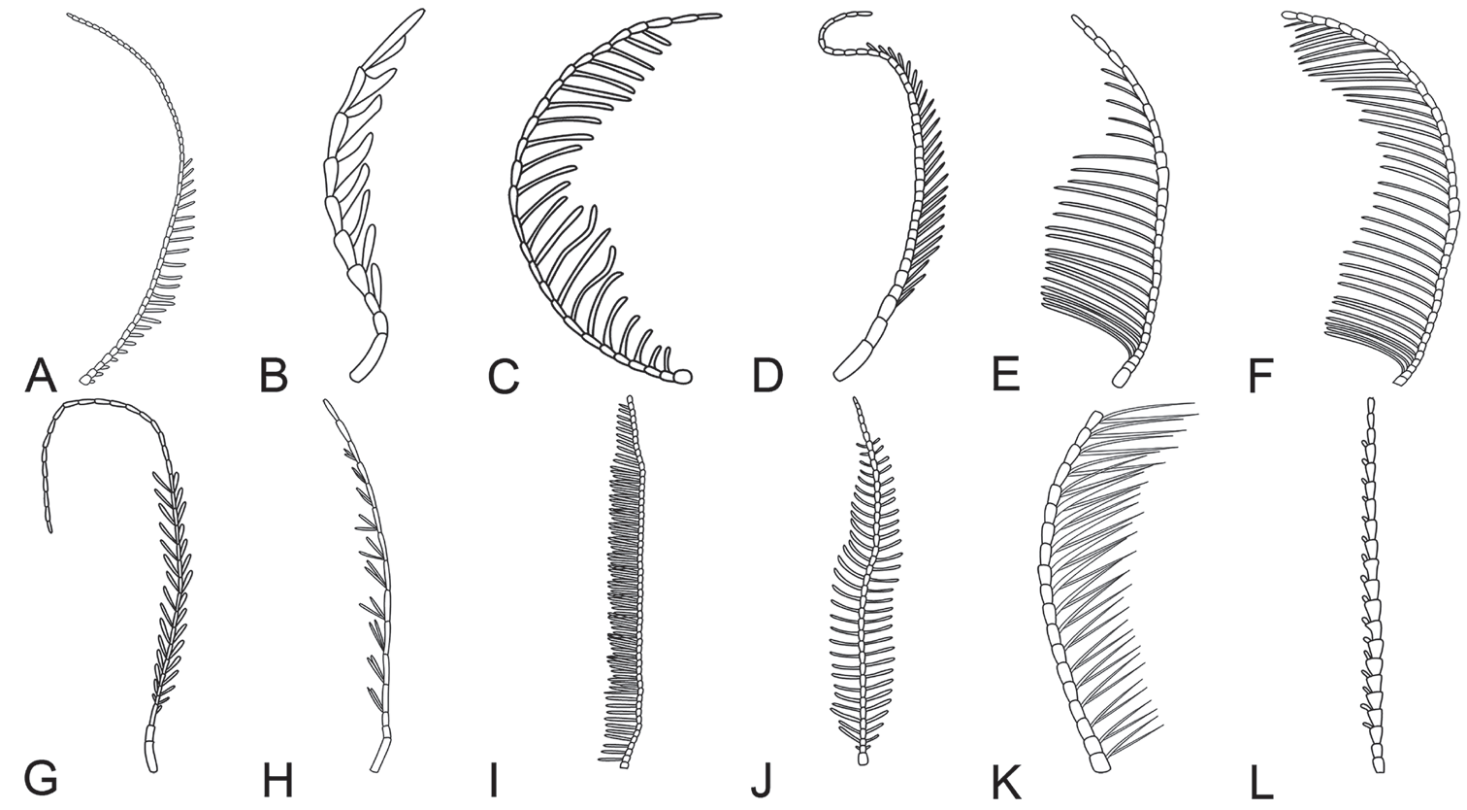
Line drawings of ramified antennae from insects of different orders **A** the pectinate antenna of Mecoptera (*Vitimopsychepectinella*) **B** the pectinate antenna of Coleoptera (*Cerophytumalbertalleni*) **C** the pectinate antenna of Neuroptera (*Cretodilarburmanus*) **D** the pectinate antenna of Hymenoptera (*Jibaissodespeichenae*) **E** the plumose antenna of Hymenoptera (*Jibaissodesbellus*) **F** the flabellate antenna of Hymenoptera (*Atefiarasnitsyni*) **G** the bipectinate antenna of Trichoptera (*Bipectinataorientalis*) **H** the bipectinate antenna of Trichoptera (*Palaeopsilotretaburmanica*) **I** the bipectinate antenna of Trichoptera (*Cathayamodusfournieri*) **J** the bipectinate antenna of Neuroptera (*Cretogrammaengeli*) **K** the bipectinate antenna of Blattodea (*Olxiai*) **L** The pectinate antenna of Blattodea (*Fragosublattapectinata* gen. et sp. nov.).

The new genus and species, *Fragosublattapectinata* gen. et sp. nov., displays distinctive comb-like extensions of pectinate antennae. This antennal modification of comb-like extensions also occurs among Cretaceous fossils of other insect orders, such as Trichoptera, Mecoptera, Hymenoptera, Coleoptera and Neuroptera (Table 2, Fig. 5). Nevertheless, there are some differences in the number and the length of comb-like extensions of pectinate or bipectinate flagellomeres. Other than the fossil insect orders mentioned above, pectinate or bipectinate antennae are known in extant insect orders, for example, Diptera (Keroplatidae, Ditomyiidae), Lepidoptera (Lymantridae, Saturniidae, etc.) and Megaloptera (Corydalidae) (Ševčík 2000; Tegoni et al. 2004; Liu and Yang 2006; Symonds et al. 2011; Ševčík et al. 2015). This new finding of pectinate antennae for a cockroach in the mid-Cretaceous, in conjunction with the other 26 fossil insects in six orders (Table 2), provides further evidence to support structural convergent evolution for ramified antennae among different insect lineages. The most direct effect of the ramified antennal structure to enhance insect sensing is the overall expansion of the antenna surface area and the corresponding increase in the number of receptors (Gao et al. 2016). Since there are only two reported male cockroaches with pectinate or bipectinate antennae, potential sexual dimorphism for mid-Cretaceous cockroaches is suggested, pending future reports of more examples and conspecific females.

The fore tibia spurs of the new species have serrations on their inner surface, which is special among cockroaches (Fig. 4B). To our best knowledge, only *Nodosigaleaburmanica* (Corydiidae) possesses similar serrations in Burmese amber (Li and Huang 2018). Besides, the tarsal plantulae in fore and mid legs are usually considered as adhesive devices allowing the cockroach to perch or forage on leaves, while the tarsal spines on hind legs are supposed to help the cockroach with rapid movement (Bell et al. 2007).

In addition, the venation and cercus of the new species are also interesting. In the right forewing, there are two incomplete CuA and A (Fig. 2C). This character has been reported in the Raphidiomimidae (Liang et al. 2009). It is likely that this phenomenon was due to the fusion of veins. The basal part of cercus for this new species is cylindrical while the terminal part is moniliform. The function or derivation of this structure of the cercus are unknown, pending future research with new fossil specimens.

## Conclusions

This study documents and reports a new species of cockroach, *Fragosublattapectinata* gen. et sp. nov., assigned to the Corydiidae. The pectinate antennae of this new species have been compared to 26 other ramified antennal structures in six orders of insects in the Cretaceous. This finding enriches the diversity of morphological characters of cockroaches and suggests that some extinct representatives of this family might have had sexual dimorphism in their antennae. Furthermore, diversified structures of ramified antennae in different orders of fossil insects during the Cretaceous provide further evidence supporting the convergent evolution of antennal structures among different insect lineages.

## Supplementary Material

XML Treatment for Fragosublatta

XML Treatment for Fragosublatta pectinata
